# Synthesis and Regioselective Reaction of Some Unsymmetrical Heterocyclic Chalcone Derivatives and Spiro Heterocyclic Compounds as Antibacterial Agents

**DOI:** 10.3390/molecules201219827

**Published:** 2015-12-10

**Authors:** Maher A. El-Hashash, Sameh A. Rizk, Saad R. Atta-Allah

**Affiliations:** Chemistry Department, Science Faculty, Ain-Shams University, Abassia, Cairo 11566, Egypt; maeelhashash@yahoo.com (M.A.E.-H.); saadrm1964@yahoo.com (S.R.A.-A.)

**Keywords:** 4-aryl-4-oxo-but-2-enoic acid, oxirane, chalcone, imidazo[2,1-*b*]thiadiazole, spiropyrazole, spiroisoxazole, spiropyrane

## Abstract

A number of novel heterocyclic chalcone derivatives can be synthesized by thermal and microwave tools. Treatment of 4-(4-Acetylamino- and/or 4-bromo-phenyl)-4-oxobut-2-enoic acids with hydrogen peroxide in alkaline medium were afforded oxirane derivatives **2**. Reaction of the epoxide **2** with 2-amino-5-aryl-1,3,4-thiadiazole derivatives yielded chalcone of imidazo[2,1-*b*]thiadiazole derivative **4** via two thermal routes. In one pot reaction of 4-bromoacetophenone, diethyloxalate, and 2-amino-5-aryl-1,3,4-thiadiazole derivatives in MW irradiation (W 250 and T 150 °C) under eco-friendly conditions afforded an unsuitable yield of the desired chalcone **4d**. The chalcone derivatives **4** were used as a key starting material to synthesize some new spiroheterocyclic compounds via Michael and aza-Michael adducts. The chalcone **4f** was similar to the aryl-oxo-vinylamide derivatives for the inhibition of tyrosine kinase and cancer cell growth. The electron-withdrawing substituents, such as halogens, and 2-amino-1,3,4-thiadiazole moeity decreasing the electron density, thereby decreasing the energy of HOMO, and the presence of imidazothiadiazole moiety should improve the antibacterial activity. Thus, the newly synthesized compounds were evaluated for their anti-bacterial activity against (ATCC 25923), (ATCC 10987), (ATCC 274,) and (SM514). The structure of the newly synthesized compounds was confirmed by elemental analysis and spectroscopic data.

## 1. Introduction

The anti-proliferative activity of (*E*)-4-aryl-4-oxo-2-butenoic acid amides was shown against three human tumor cell lines [1], in addition to a multitude of biological activities [2]. Chalcone derivatives are one of the major classes of natural products with widespread distribution in fruits, vegetables, spices, tea and soy based foodstuff. Recently, they have been a subject of great interest for their interesting pharmacological activities [3]. A series of chalcone derivatives bearing heterocycles [4] and synthesis of the heterocyclic chalcone in combination with antibiotics [5] were recorded. Most of the chalcones are highly biologically active with a number of pharmacological and medicinal applications [6]. Spiroindoline [7] and imidazoline derivatives [8] can be evaluated for their binding affinities and antagonistic activities at the neuropeptide YY5 receptor, as well as their good brain penetration. Also, spironolactone [9,10] is effective in treating mild hypertension without inducing hypokalemia or increased secretion of Aldosterone and Ephlerenone. Notably, ketoconazole [11,12] has been successful as an antifungal agent. If the spiroimidazole derivatives [13] are combined with antibacterial agents (vancomycin, ciprofloxacin), it may be observed that antagonistic activity results from the competitive binding of the medicinal molecules into bacteria cells’ receptor. On the other hand, the isoxazolines [14,15] are evaluated for their *in vitro* antifungal activity and their proliferative response to human mononuclear peripheral blood cells. Imidazo-oxazole derivatives [16] can be synthesized via treatment of imidazole derivatives with oxirane, and they have been tested for anti-mycobacterial activity. The (*E*)-4-aryl-4-oxo-2-butenoic acids are convenient poly electrophilic reagents for the addition reaction of nucleophiles; e.g., carbon, nitrogen, and sulfur occur exclusively at the α-carbon electrophilic center of the carboxy precursors used for the synthesis of relevant heterocyclic compounds [17,18,19,20,21,22]. The authors have reported [19,23] that the behavior of 4-(4-acetyl amino/bromo phenyl)-4-oxo-but-2-enoic acids (**1**) toward the hydrogen peroxide in the presence of 8% sodium hydroxide/methanol afforded the epoxide products of (*E*)-1-(4-acetylaminobenzoyl)-2-oxirane carboxylic acids (**2**). Among them, imidazo[2,1-*b*]1,3,4-thiadiazole is an attractive aryl unit that causes a decrease in electron density (low HOMO) of the synthesized chalcones, which increases its antibacterial activity [24].

## 2. Results and Discussion

### 2.1. Chemistry

**Scheme 1:**
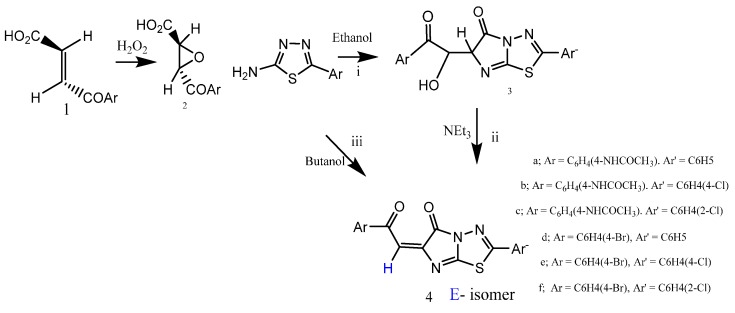
Synthetic routes for compounds **2**–**4**. (i) Reaction of the oxirane derivative **2** within 2-amino-1,3,4-thiadiazole in boiling ethanol afforded α-hydroxy ketone **3**; (ii) Refluxing the derivatives **3** in boiling ethanol/TEA, afforded chalcone derivatives **4** in good yield 60%–71%; (iii) Direct synthesis of the chalcone derivatives **4** in butanol/reflux 5 h, but in poor yield of 35%–40%.

The regioselective reaction of (*E*)-1-(aroyl)-2-oxirane carboxylic acids (**2**) with 2-amino-5-aryl-1,3,4-thiadiazole in the presence of boiling ethanol afforded imidazo[2,1-*b*]thiadiazole derivatives **3** [18,19], via the *N*-alkylation of amino thiadiazole moieties that added to the activated α- position of 3-membered heterocycle [25] of the carboxylic acids **2** (Scheme 1). Refluxing adducts **3** with drops of triethylamine [TEA] in boiling ethanol afforded the chalcone derivatives **4** in good yield. The geometrical isomerism of the compounds **4** can be detected only in ^1^H-NMR spectra. The ^1^H-NMR of the compound **4a** in DMSO reveals δ ppm at 2.54 (s, CH_3_) corresponding to CH_3_CONH precursor, multiplied by 7.48–7.86 corresponding to the aromatic protons; the proton of arylidine has two chemical shift values at 7.72, 1*H*, s, CH=, arylidine, referring to the *E*-configuration, 82% (high integrated value (%) in ^1^H-NMR reflects stability of *E*-isomer), and at 7.78, 1*H*, s, CH=, arylidine, 18% in the form of a *Z*-configuration. The chemical shift δ ppm of the arylidine proton of the *Z*-configuration was increased due to the field effect of the carbonyl moiety of the imidazole, and at 13.2, s, acidic NH proton was exchangeable with D_2_O.

**Scheme 2:**
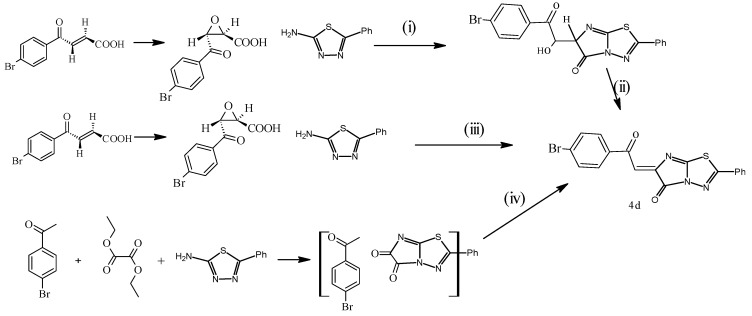
Synthetic route for compound **4d**. Reagents and Conditions: (i) ethanol/reflux 3 h, 74%; (ii) ethanol/TEA/reflux 5 h, good yield 60%–71%; (iii) Butanol/reflux 5 h, with a poor yield of 35%–40%; (iv) MW irradiation (W 250 and T 150 °C) for 15 min, with a poor yield of 35%–45%.

An authentic reaction was done, when the acid **2** was submitted to react with 2-amino-5-aryl-1,3,4-thiadiazole in boiling butanol, which led to spontaneous dehydration of the adduct **3** to afford the more thermodynamically stable **4** (Scheme 1). The new chalcone product **4**, the thermally labile acid **2** and/or substituted aminothiazoles were very sensitive to higher temperatures. The classical synthesis (Route iii) in Scheme 1 and Scheme 2 was not suitable because the prolonged (4–6 h) heating (110–120 °C) led to butanol yields lower than 40% and which tended to decrease. The novel chalcone derivative **4** can also be synthesized in one pot reaction, fusing the 2-aminothiadiazole, diethyloxalate and 4-bromoacetophenone derivatives in pellet KOH, with a small amount of water (useless organic solvent) in MW irradiation (W 250 and T 150 °C) for 15 min, in line with eco-friendly environmental chemistry, afforded the chalcone derivative **4d**, but also, it was formed with a poor yield of 40%. Scheme 2 outlines the synthesis of the novel chalcone derivative **4d**. Attempts to have favourable access to the desired chalcone using suitable base triethylamine [TEA] led to the decomposition of the reactive chalcone **4**, when the authors used strong basic medium.

**Scheme 3:**
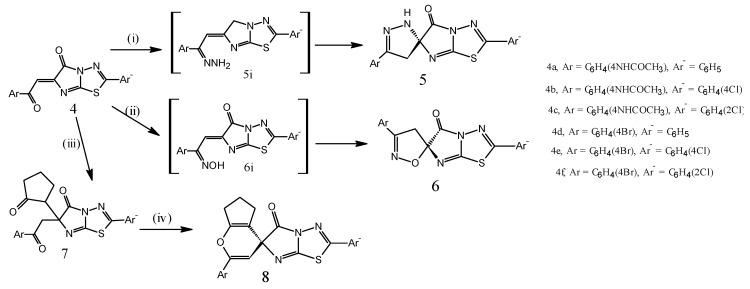
Synthetic route for compounds **5**–**8**. Reagents and Conditions: (i) NH_2_NH_2_/ethanol/reflux 6 h, 60%–65%; (ii) NH_2_OH/pyridine/reflux 5 h, 55%–70%; (iii) Cyclopentanone/ethanol/NaOH(50%)/Stir, 65%–74%; (iv) Acetic anhydride/ reflux 1 h, 35%–45%.

Chalcone bears a very good synthon so that varieties of novel heterocycles with good pharmaceutical profile can be designed [4,5,6]. An interesting feature of this structure is a pincer-like conformation of the molecule [26], and a reaction between isatin and the α-amino acid afforded the azomethine ylide, with regioselective addition to the C=C bond of aroylacrylic acid or chalcone. The electrophilic centers in **4** can be allowed to react with simply bi-nucleophiles, e.g., hydrazine derivatives and hydroxyl amine, to afford important spiro heterocyclic compounds [19]. Treatment of the isomers **4b**, **4d**, **4e** and **4f** with hydrazine hydrate and/or hydroxylamine (Scheme 3) afforded spiro heterocyclic compounds **5** and **6** via formation of the hydrazone and oxime intermediates **5i** and **6i**, respectively [19]. Moreover, when the chalcone derivatives **4** were allowed to react with the cyclopentanone in the presence of sodium hydroxide, this afforded 50% adducts **7** [27]. Treatment of the adduct **7** with acetic anhydride afforded spiro-pyrane derivative **8** instead of formation of the furo[3,2-*d*]1,3,4-thiadiazole[3,2-*a*]imidazole [19]. These reactions can be reflected in the reactivity of the carbonyl group of the aroyl moiety which is greater than the carbonyl group of imidazole moiety. The authors reported that the product **8** can be changed and returned to **7** after approximately one day. The lower stability of the product **8** allowed the ring to open again and return to the product **7** due to the bridgehead spiro carbon atom that can be surrounded by four *sp*^2^ atoms.

The procedure would include arrest of the reaction at the cycloalkane level and restart with different carbon nucleophiles. The authors expected that in the case of electron withdrawing groups in the pyrane structures, the best yields were attained by the direct procedure. As it is shown, the best yield in spiro-pyrane derivative **8** were achieved for the derivative **8a**, which subsequently its chalcone **4a** was the most commonly used in the continued research.

**Scheme 4:**
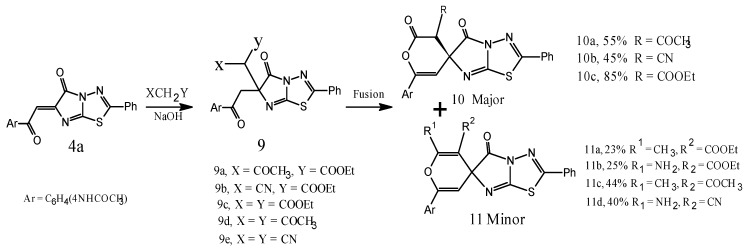
Synthetic route for compounds **9**–**11**.

So, when the chalcone **4a** was allowed to react with different carbon acids, e.g., ethylacetoacetate, ethylcyanoacetate, diethylmalonate, acetylacetone, and malononitrile. The reaction can proceed with ethylacetoacetate through the intermediate **9a** followed by ring closure via the tetrahedral mechanism to afford the regioselective product **10** that can be confirmed by the lower stability of the product **11** (Scheme 4). The authors assumed this one-pot reaction would similarly work with ethylcyanoacetate and diethylmalonate that confirmed this fact through the cyclization of the intermediates **9b**–**c**. In the same manner, the spiro-pyrane derivative **11** was alternatively substituted with R^1^ and R^2^ groups, they were synthesized in good yields from acetylacetone and/or malononitrile (the same bi-functional carbon acids). According to the proposed pathway, the use of a controlled sequential procedure for the preparation of spiro-pyrone **10** and spiro-pyrane **11** should give access to a wider series of spiro compounds **10** and **11**, having different substituents R, R^1^, and R^2^.

### 2.2. Antibacterial Activity Evaluation

#### Agar Diffusion Method

The obtained new compounds were screened *in vitro* for their antibacterial activities against Gram positive bacteria (*Staphylococcus aureus* (ATCC 25923) and *Bacillus cereus* (ATCC 10987)), Gram negative bacteria (*Serratia marcesens* (ATCC 274) and *Proteus mirabilis* (SM514)), using the agar diffusion technique. The results of the antibacterial activity tests are shown in Table 1.

**Table 1:** Antibacterial activity of the synthesized compounds: Agar diffusion method.

Compound No.	Gram Positive		Gram Negative	
	*Staphylococcus aureus*	*Bacillus cereus*	*Serratia marcesens*	*Proteus mirabilis*
**4a**	+++	++	+++	++
**4b**	+++	++	++	++
**4c**	+++	+++	+++	++
**4d**	+++	++	++	++
**4e**	++	+	+	+
**4f**	+++	+++	++	+++
**6a**	++	++	++	+
**6b**	++	++	++	+
**7a**	++	++	+++	+
**7b**	++	++	+++	+
**8a**	−	+	−	−
**8b**	+	+	+	+
**9a**	++	++	+++	+++
**9b**	++	+++	+++	++
**10a**	+	+	+	+
**10b**	+	+	+	+
Chloramphenicol^®^	+++	+++	+++	+++
Ampicillin^®^	+++	+++	+++	+++

The width of the zone of inhibition indicates the potency of antibacterial activity; (−) no antibacterial activity (0%–25%); (+) mild activity with the diameter of the zones equal to 0.5–0.8 cm (25dehydroascorbate 40%); (++) moderate activity with the diameter of the zones equal to 1.1–1.2 cm (55%–65%); (+++) marked high activity with the diameter of the zones equal to 1.8–2.0 cm (85%–100%).

Most of the synthesized compounds were found to possess some antibacterial activity towards all the microorganisms used. Compounds **4**, **6**, **7**, **9** possess the highest antibacterial activities because they have been 65%–95% inhibition zone for antibacterial activity for both gram positive and gram negative bacteria.

**Figure 1:**
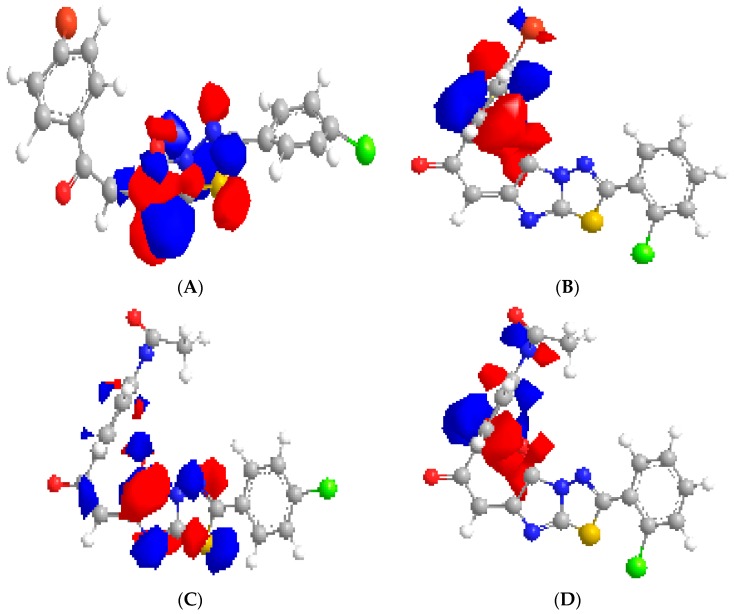
Outlines the electron distribution in HOMO for **4e** (**A**); **4f** (**B**); **4b** (**C**) and **4c** (**D**) compounds, respectively, are outlined, confirming that electron density is low among the o-chloro derivatives (decreased HOMO and increased activity). Electron deficient at the center of molecule means increase the antibacterial activity as the compounds **4f** (**B**) and **4c** (**D**).

**Figure 2:**
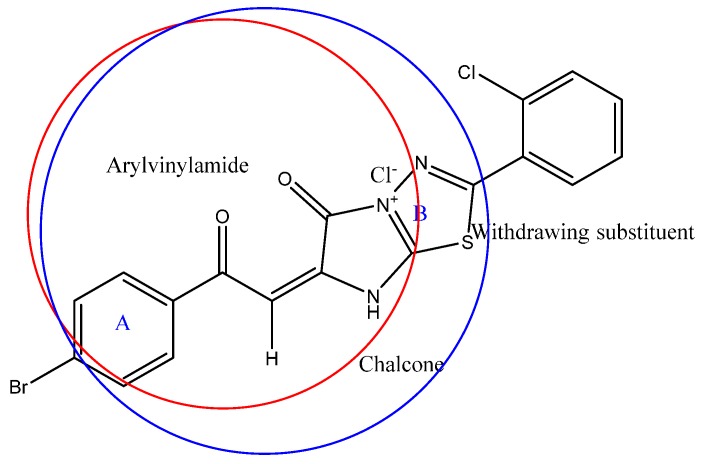
Outlines the ring B in a blue circle that contains three electronegative nitrogen atoms, a carbonyl group and a halogen atom. These elements decrease the HOMO value and increase activity. Red circle outline aryl vinyl amide structure isostere for synthesized chalcones **4**.

The generated QSAR model [24] indicates that a minimum HOMO energy of more than 30 chalcone derivatives contributes positively to the antibacterial activity. Electron-withdrawing substituents are lower the HOMO energy, such as halogens, due to the inductive effect of halogen which results in the decrease in electron density from the σ space of benzene ring, particularly o-chloro derivatives, thereby decreasing the energy of HOMO [28]. Designing chalcone derivatives with a high degree of bonding linearity (κ2 index) with groups that increase molecular weight (high value of ADME Weight) represents a positive contribution to the antibacterial activity [24]. P-glycoprotein (P-gp) is an ATP-dependent multidrug resistance efflux transporter that plays an important role in anticancer drug resistance and in the pharmacokinetics of medicines [29]. The bio-isostere of aroyl vinylamide and the new synthetic compound **4** indicated antitumor activities, as well as tyrosine kinase inhibition [30]. So, the authors wanted to synthesize unsymmetrical heterocyclic chalcone derivatives possessing imidazo-thiadiazole moiety, considered as antibacterial agents [16], with high molecular weight and electron withdrawing groups (low HOMO values); e.g., o-halo aryl and 2-amino-1,3,4-thiazole precursors and the characteristic linearity of bonding patterns (high κ2) that exhibit high antibacterial activity, *c.f.* Table 1 and Table 2 and Figure 1 and Figure 2. The authors explained that the strongest activities of the synthetic compounds **4c** and **4f** (Table 1) were due to the inductive effect of the 2-chloro derivatives that decreases the electron density (decreased HOMO values), as shown in Figure 1B,D and increases the antibacterial activity. But the 4-chloro derivatives have electromeric effects that increase the electron density (increased HOMO) and decrease activity, as shown in Figure 1A,C. Also, the results are shown in Table 2, screen the Minimum Inhibitory Concentration (MIC) and calculated values of ADME, HOMO, and κ2 that are used to generate the QSAR model. The effect of amino-1,3,4-thiazole precursors were stronger than pyridyl and nitrophenyl precursors [24] that outlined the strong antibacterial activity of the synthesized compounds (Table 2).

On the other hand, the resistance mechanism to penicillin antibiotics in these bacteria is the expression of beta-Lactamase enzyme. In order to use the penicillin antibiotics which are still effective against them, Jaramillo *et al.* [31] had evaluated many chalcones as inhibitors of this enzyme. The chalcone derivatives **4** exhibit high antibacterial activity due to the presence of activated double bond as capping agent for the enzyme, means the chalcones as a possible drug (enzyme inhibitor). Also, the spiro compounds **6** exhibit high antibacterial activity [32,33,34] as compared with compounds **5** and **10**, because of the spiro five membered rings is near to structure of penicillin core (β-lactam-thiazole ring) and so they can be matching with the enzyme more than compounds **10**. Compounds **5** have an acidic NH group of pyrazole precursor and so they have less fitting with enzyme. On the other hand, the compounds **7**, and **9** exhibit high antibacterial activity due to the presence of the carbonyl groups that condensed with NH_2_-E (enzymatic inhibitor).

**Table 2:** Rationalization of the synthesized Chalcones **4** as antibacterial agents using quantum chemical computation.

Comp. Ref.	Substituent Ring A	Substituent Ring B	MIC ^a^ (ug/mL)	ADME ^b^ Weight	HOMO ^b^	κ2 Index ^b^
**4a**	NHCOCH_3_	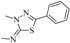	600	320.3	−11.864	8.762
**4b**	NHCOCH_3_	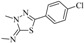	700	253.6	−10.282	9.163
**4c**	NHCOCH_3_	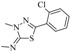	500	393.1	−13.409	9.718
**4d**	Br	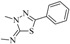	600	314.3	−11.940	7.415
**4e**	Br	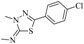	700	225.2	−11.322	7.505
**4f**	Br	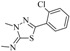	500	275.3	−13.918	8.914
**6** [24]	H	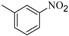	600	321.3	−9.752	8.590
**9** [24]	H	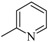	700	277.29	−9.509	7.513

^a^ Minimum Inhibitory Concentration; ^b^ Calculated values used to generate QSAR models.

## 3. Experimental Section

### 3.1. General Information

All melting points are corrected and determined on a stuart electric melting point apparatus (Microanalytical centre, ainshams university, Cairo, Egypt). Elemental analyses were carried out by Elementar Viro El-Microanalysis at the Micro-analytical Center, National Research Center, Egypt. IR spectra (KBr) were recorded on infrared spectrometer FT-IR 400D (New York, NY, USA) using OMNIC program and are reported frequency of absorption in terms of cm^−1^ and ^1^H-NMR spectra recorded on a Bruker spectrophotometer (Rheinstetten, Germany) at 400 MHz using TMS as internal standard and with residual signals of the deuterated solvent δ = 7.26 ppm for CDCl_3_ and δ 2.51 ppm for DMSO-*d*_6_. ^13^C-NMR spectra were recorded on the same spectrometer (Rheinstetten, Germany) at 100 MHz and referenced to solvent signals δ = 77 ppm for CDCl_3_ and δ 39.50 ppm for DMSO-*d*_6_. DEPT 135 NMR spectroscopy were used where appropriate to aid the assignment of signals in the ^1^H- and ^13^C-NMR spectra. The mass spectra were recorded on Shimadzu GCMS-QP-1000 EX mass spectrometer (Kyoto, Japan) used the electron ionization technique at 70 e.v. Homogeneity of all synthesized compounds was checked by TLC.

### 3.2. General Procedure for Synthesis of the Compounds ***2***, ***3***, ***5a*** and ***5c*** are in the Literature [19]

### 3.3. General Procedure for Synthesis of the Compounds ***4a***–***f***

Fuse the compounds **3a**–**f** (0.01 mol) and 3–5 drops triethyl amine (TEA) in oil bath for 5 min, then refluxing in 50 mL of boiling aqueous ethanol for 5 h. The solid was separated after cooling and the pH of the solution was 6.5. The crude products were filtered, washed by petroleum ether (b.p. 40–60 °C), dried and then recrystallized from dioxane.

*(E)-N-(4-(2-(5-Oxo-2-Phenylimidazo[2,1-b]1,3,4-thiadiazol-6(5H)ylidine)acetyl)phenyl)acetamide* (**4a**). Yield 2.35 g (60%), light yellow finely crystalline, m.p. 176–178 °C. IR (KBr), υ, cm^−1^: 3245 (NH), 3055 (CH), 1706, 1670, 1650 (CO), 1613 (C=N). ^1^H-NMR (DMSO-*d*_6_), δ, ppm, (*J*, Hz): 2.54 (3H, s, CH_3_), 7.72 (1H, s, CH=, arylidine, 82% in form of *E*-configuration), 7.78 (1H, s, CH=, arylidine, 18% in form of *Z*-configuration), 7.48–7.86 (9ArH, m, aromatic protons), 13.2 (1H, s, acidic NH proton which exchanged in D_2_O), ^13^C-NMR δ, 40.0 (CH_3_CO), 105.4 (C_2,6_ Ph), 110.6 (C_3,5_ Ph), 125.5 (C_4_ Ar), 142.3 (C_3,5_ Ar), 144.8 (CH=), 147.4 (C_2_ Ar), 148 (C_6_ Ar), 148.5 (C_4_ Ph), 150.2 (C=CH), 152.4 (C_1_ Ph), 154.5 (C_1_ Ar), 156.0 (CNS), 162.2 (CN_2_S), 165.1 (CO imidaz.), 168.0 (CO amide), 190.2 (CO ketone), and found, %: C 61.50, H 3.59, N 14.30, S 8.19 for C_20_H_14_N_4_O_3_S. Calculated, %: C 61.53, H 3.61, N 14.35, S 8.21; MS: *m*/*z* 346 [M^+^ − CH_2_=C=O], 141 [imidazole thiadiazole moiety].

*(E)-N-(4-(2-(2-(4-Chlorophenyl-5-oxo-imidazo[2,1-b]1,3,4-thiadiazol-6(5H)ylidine)acetyl)phenyl)acetamide* (**4b**). Yield 2.85 g (67%), yellow finely crystalline, m.p. 210–212 °C. IR (KBr), υ, cm^−1^: 3245 (NH), 1710, 1691, 1655 (CO); 1630 (C=N); ^1^H-NMR (DMSO-*d*_6_), δ, ppm, (*J*, Hz): 2.06 (3H, s, CH_3_), 7.73 (1H, s, CH=, arylidine, 78% in form of *E*-configuration), 7.81 (1H, s, CH=, arylidine, 22% in form of *Z*-configuration), 7.44–7.83 (8ArH, m, aromatic protons), 12.6 (1H, s, acidic NH proton which exchanged in D_2_O); ^13^C-NMR δ 22.5 (CH_3_CO), 121.2 (C_2,6_ Ph), 128.8 (C_3,5_ Ph), 129.5 (C_4_ Ar), 131.5 (C_4_ PhCl), 132.3 (C_3,5_ Ar), 134.6 (CH=), 137.1 (C_2_ Ar); 138.3 (C_6_ Ar), 140.2 (C=CH), 142.0 (C Ph), 142.4 (C_1_ Ar), 160.3 (CNS), 161.4 (CN_2_S), 166.5 (CO imidaz), 167.4 (CO amide), 191.0 (CO ketone) and found, %: C 56.53; H 3.06; N 13.16, Cl 8.30, S 7.53 for C_20_H_13_N_4_O_3_SCl. Calculated, %: C 56.54, H 3.08, N 13.19, Cl 8.34, S 7.55.

*(E)-N-(4-(2-(2-(2-Chlorophenyl-5-oxo-imidazo[2,1-b]1,3,4-thiadiazol-6(5H)ylidine)acetyl)phenyl)acetamide* (**4c**). Yield 2.68 g (63%), yellow finely crystalline, m.p. 198–200 °C. IR (KBr), υ, cm^−1^: 3245 (NH); 1710, 1691, 1655 (CO); 1630 (C=N). ^1^H-NMR (DMSO-*d*_6_), δ, ppm, (*J*, Hz): 2.06 (s, 3H, CH_3_), 7.69 (1H, s, CH=, arylidine, 80% in form of *E*-configuration), 7.77 (1H, s, CH=, arylidine, 20% in form of *Z*-configuration), 7.44–7.83 (8ArH, m, aromatic protons), 12.4 (1H, s, acidic NH proton which exchanged in D_2_O), and found, %: C 56.50, H 3.02, N 13.11, Cl 8.30, S 7.51 for C_20_H_13_N_4_SO_3_Cl. Calculated, %: C 56.54, H 3.08, N 13.19, Cl 8.34, S 7.55; MS: *m*/*z* 389 [M^+^ − Cl], 347 [389 − CH_2_CO].

*(E)-6-(4-Bromophenyl)-2-oxoethylidine)-2-phenylimidazo[2,1-b]1,3,4-thiadiazol-5(6H)-one* (**4d**). Yield 2.72 g (66%), yellow powder, m.p. 152–154 °C. IR (KBr), υ, cm^−1^: 1694, 1672 (CO); 1613 (C=N). ^1^H-NMR (DMSO-*d*_6_), δ, ppm, (*J*, Hz): 7.70 (1H, s, CH=, arylidine, 86% in form of *E*-configuration), 7.79 (1H, s, CH=, arylidine, 14% in form of *Z*-configuration), 7.44–7.73 (9ArH, m, aromatic protons). ^13^C-NMR δ 119.6 (C_2,6_ Ph), 127.6 (C_3,5_ Ph), 130.5 (C_4_ Ph), 132.7 (C_3,5_ Ar), 135.3 (C_2,6_ Ar), 137.6 (C_1_ Ph), 139 (CH=), 141.2 (C_4_ Ar), 142.1 (C_1_ Ar), 145.5 (C=), 149.7 (CNS), 155.3 (CN_2_S), 166.8 (CO imidaz.), 179.2 (CO) and found, %: C 52.50; H 2.35; N 10.15. C_18_H_10_N_3_O_2_BrS. Calculated, %: C 52.42, H 2.42, N 10.19. MS: *m*/*z* 378 [M^+^ − CO], 335 [M^+^ − Ph], 263 [M^+^ − Ph(Br)], 138 [imidazolothiadiazole moiety].

*(E)-6-(4-Bromophenyl)-2-oxoethylidine)-2-(4-chlorophenyl)imidazo[2,1-b]1,3,4-thiadiazol-5(6H)-one* (**4e**). Yield 3.17 g (71%), yellow finely crystalline, m.p. 168–170 °C. IR (KBr), υ, cm^−1^: 1710, 1691 (CO), 1630 (C=N); ^1^H-NMR (DMSO-*d*_6_), δ, ppm (*J*, Hz): 7.70 ( 1H, s, CH=, arylidine, 78% in form of *E*-configuration), 7.80 (1H, s, CH=, arylidine, 22% in form of *Z*-configuration), 7.60–7.83 (8ArH, m, aromatic protons), and found, %: C 48.30, H 2.10, N 9.33. C_18_H_9_N_3_O_2_BrClS. Calculated, %: C 48.37, H 2.15, N 9.40. 

*(E)-6-(4-Bromophenyl)-2-oxoethylidine)-2-(2-chlorophenyl)imidazo[2,1-b]1,3,4-thiadiazol-5(6H)-one* (**4f**). Yield 3.04 g (68%), yellow finely crystalline, m.p. 180–182 °C. IR (KBr), υ, cm^−1^: 1708, 1684 (CO); 1630 (C=N). ^1^H-NMR (DMSO-*d*_6_), δ, ppm, (*J*, Hz): 7.73 (1H, s, CH=, arylidine, 73% in form of *E*-configuration), 7.84 (1H, s, CH=, arylidine, 27% in form of Z-configuration), 7.72–7.86 (8ArH, m, aromatic protons), and found, %: C 48.35, H 2.15, N 9.35 for C_18_H_9_N_3_O_2_BrClS. Calculated, %: C 48.37; H 2.15; N 9.40.

### 3.4. General Procedure for Synthesis of the Compounds ***5b***, ***5d***, and ***5e***

A mixture of chalcone derivatives **4b**, **4d** and **4e** (5 mmol) and hydrazine hydrate (0.5 mL, 0.01 mol) in boiling ethanol (50 mL) was heated under reflux for 5 h. The reaction mixture was allowed to cool and the product was filtered, dried, and recrystallized from the benzene and/or ethanol.

*N-(4-(2-(2-Chlorophenyl)-5-oxo)-2′,4′-dihydro-5H-spiro[imidazo[2,1-b]1,3,4-thiadiazol-6,3′-pyrazol]-5′yl) phenyl)acetamide* (**5b**). Yield 1.43 g (65%), off white crystal, m.p. 164–166 °C. IR (KBr), υ, cm^−1^: 3420 (NH); 1671, 1640 (CO); ^1^H-NMR (DMSO-*d*_6_), δ, ppm (*J*, Hz): 1.2 (d, 2H, CH_2_, *J* = 5.4); 2.5 (3H, s, CH_3_); 5.8 (1H, br. s, NH); 7.0–7.81 (8H, m, Ar-H); 12.40 (1H, br. s, NH of acetamide moiety), ^13^C-NMR δ, 22.3 (CH_3_CO); 69.6 (CH_2_C=N); 122.4 (C_4_ Ph), 126.8 (C_3,5_ Ph), 129.1 (C Spiro), 131.5 (C_3_ Ar), 131.8 (C_5_ Ar), 132.6 (C_2,6_ Ph), 137.4 (C_2_ Ar), 138 (C_6_ Ar), 140.2 (C_4_ Ar), 144.5 (C_1_ Ar), 145.1 (C_1_ Ph), 158.0 (C=N), 161.3 (CNS), 163.2 (CN_2_S), 165.3 (CO imidaz.), 167.8 (CO amide), and found, %: C 54.70, H 3.42, N 19.10, Cl 8.02, S 7.27 for C_20_H_15_N_6_O_2_ClS. Calculated, %: C 54.73, H 3.45, N 19.15, Cl 8.08, S 7.30. 

*5′-(4-Bromophenyl)-2-phenyl-2′,4′-dihydro-5H-spiro[imidazo[2,1-b]1,3,4-thiadiazol-6,3′-pyrazol]-5-one* (**5d**). Yield 1.28 g (60%), White finely crystalline, m.p. 138–140 °C. IR (KBr), υ, cm^−1^: 3423, 3151 (NH), 1671, 1640 (CO), ^1^H-NMR (DMSO-*d*_6_), δ, ppm, (*J*, Hz): 1.2 (2H, d, CH_2_, *J* = 5.5), 2.5 (3H, s, CH3), 5.4 (1H, s, NH), 7.0–7.81 (9H, m, Ar-H); ^13^C-NMR δ, 69.4 (CH_2_C=N), 122.1 (C_4_ Ph), 125.9 (C_3,5_ Ph), 127.1 (C Spiro), 131.4 (C_3_ Ar), 131.8 (C_5_ Ar), 132.5 (C_2,6_ Ph), 136.8 (C_2_ Ar), 137.4 (C6 Ar), 140.2 (C4 Ar), 144.5 (C_1_ Ar); 145.1 (C_1_ Ph), 158.0 (C=N), 161.3 (CNS), 163.2 (CN_2_S); 165.3 (CO imidaz.), and found, %: C 50.70, H 2.81, N 16.40, Br 18.71, S 7.50 for C_18_H_12_N_5_OBrS. Calculated, %: C 50.72, H 2.84, N 16.43, Br 18.74, S 7.52.

*5′-(4-Bromophenyl)-(2-(4-chlorophenyl)-2′,4′-dihydro-5H-spiro[imidazo[2,1-b]1,3,4-thiadiazol-6,3′-pyrazol]-5-one* (**5e**). Obtained similarly to compound **5a** from compound **4f** (2.23 g, 5 mmol). Yield 1.50 g (65%), white finely crystalline, m.p. 150–152 °C. IR (KBr), υ, cm^−1^: 3423, 3333 (NH); 1680, 1648 (CO); ^1^H-NMR (DMSO-*d*_6_), δ, ppm, (*J*, Hz); 1.4 (2H, d, CH_2_, *J* = 5.4), 2.5 (3H, s, CH3), 5.4 (1H, s, NH), 7.0–7.81 (8H, m, Ar-H); found, %: C 46.89, H 2.40, N 15.20, Br 17.32, Cl 7.67, S 6.92 for C_18_H_11_N_5_OBrClS. Calculated, %: C 46.92, H 2.41, N 15.20, Br 17.34, Cl 7.69, S 6.95; MS: *m*/*z* 464 [M^+^ + 2]; 461 [M^+^]; 418 [M^+^ − CH_2_=C=O]; 194 [spiro moiety].

### 3.5. General Procedure for Synthesis of the Compounds ***6a***–***d***

A mixture of **4a**, **4b**, **4d** and/or **4f** (5 mmol) and hydroxyl amine hydrochloride (0.52 g; 7.5 mmol) in boiling pyridine (25 mL) was heated under reflux for 6 h. The reaction mixture was allowed to cool, poured into ice/HCl until pH of the solution is 6.5, and the product was filtered, dried, and recrystallized from ethanol.

*N-(4-(5-Oxo-2-phenyl-2′,4′-dihydro-5H-spiro[imidazo[2,1-b]1,3,4-thiadiazol-6,5′-isoxazol]-3′yl)phenyl)acetamide* (**6a**). Yield 1.11g (55%), white finely crystalline. m.p. 197–200 °C. IR (KBr), υ, cm^−1^: 3425 (NH), 1647 (CO), 1630 (C=N); ^1^H-NMR (DMSO-*d*_6_), δ, ppm, (*J*, Hz): 2.5 (3H, s, CH_3_), 3.59 (2H, d, CH_2_, *J* = 5.7), 7.53–7.96 (10H, m, Ar-H), and found, %: C 59.30, H 3.65, N 17.24. C_20_H_15_N_5_O_3_S. Calculated, %: C 59.26, H 3.70, N 17.28. 

*N-(4-(2-(4-Chlorophenyl-5-oxo-2′,4′-dihydro-5H-spiro[imidazo[2,1-b]1,3,4-thiadiazol-6,5′-isoxazol]-5′yl)phenyl)acetamide* (**6b**). Yield 1.47 g (67%), white finely crystalline powder, m.p. 192–195 °C. IR (KBr) υ, cm^−1^: 3271 (NH), 1660 (CO), 1631 (C=N). ^1^H-NMR (DMSO-*d*_6_), δ, ppm, (*J*, Hz): 2.3 (3H, s, CH_3_), 3.20 (2H, d, CH2, *J* = 5.7), 7.53–7.96 (10H, m, Ar-H); ^13^C-NMR (DMSO), δ, ppm, 21.7 (CH_3_CO), 66.6 (CH_2_C=N), 119.7 (C spiro), 126.8 (C_3,5_ Ar), 128.3 (C_3,5_ PhCl), 130.5 (C_2,6_ PhCl), 134.5 (C_2,6_ Ar), 136.9 (C_4_ PhCl), 138.2 (C_4_ Ar), 140.2 (C_1_ Ar), 143.5 (C_1_ PhCl), 147.1 (C=N), 161.3 (CNS), 164.0 (CN_2_S), 166.4 (CO imidaz.), 168.0 (CO amide), and found, %: C 54.58, H 3.19, N 15.88, Cl 8.02, S 7.27 for C_20_H_14_N_5_O_3_ClS. Calculated, %: C 54.61, H 3.21, N 15.92, Cl 8.06, S 7.29.

*3′-(4-Bromophenyl)-2-phenyl-4′H,5H-spiro[imidazo[2,1-b]1,3,4-thiadiazol-6,5′-isoxazol]-5-one* (**6c**). Yield 1.56 g (70%), white finely crystalline, m.p. 197–200 °C. IR (KBr) υ, cm^−1^: 3425 (NH); 1630 (C=N). ^1^H-NMR (DMSO-*d*_6_), δ, ppm, (*J*, Hz): 3.99 (2H, s, CH_2_), 7.53–7.96 (10H, m, Ar-H). ^13^C-NMR (DMSO), δ, ppm, 65.8 (CH_2_C=N); 111.2 (C spiro); 116.2 (C_3,5_ Ar); 120.9 (C_3,5_ Ph); 124.6 (C_2,6_ Ar); 132.7 (C_2,6_ Ph); 134.3 (C_4_ Ph); 136.3 (C4 PhBr), 139.2 (C1, PhBr); 140.6 (C_1_, Ph); 153.0 (C=N); 157.2 (CNS); 162.3 (CN_2_S); 166.0 (CO imidaz.) and found, %: C 50.60, H 2.58, N 13.12, Br 18.68, S 7.48 for C_18_H_11_N_4_O_2_BrS. Calculated, %: C 50.60, H 2.60, N 13.11, Br 18.70, S 7.50. 

*3′-(4-Bromophenyl)-2-(2-chlorophenyl)-4′H,5H-spiro[imidazo[2,1-b]1,3,4-thiadiazol-6,5′-isoxazol]-5-one* (**6d**). Yield 1.39 g (58%), white finely crystalline powder, m.p. 192–195 °C. IR (KBr) υ, cm^−1^: 3271 (NH), 1680 (CO), 1631 (C=N). ^1^H-NMR (DMSO-*d*_6_), δ, ppm, (*J*, Hz): 3.49 (2H, br. s, CH_2_), 7.53–7.96 (10H, m, Ar-H), found %: C 46.85, H 2.16, N 12.11, Br 17.29, Cl 7.65, S 6.92 for C_18_H_10_N_4_O_2_BrClS. Calculated, %: C 46.82, H 2.18, N 12.13, Br 17.31, Cl 7.68, S 6.94.

### 3.6. General Procedure for Synthesis of the Compounds ***7a***–***c***

A mixture of **4a**, **4b** and/or **4d** (5 mmol), cyclopentanone (0.45 mL, 5 mmol), (50%) NaOH (5 mL) and ethanol (25 mL) was refluxed for 3 h, and left overnight for 3 days. The reaction mixture was poured into ice/HCl, until pH of the solution becomes 6.5. The crude product was filtered, washed by petroleum ether (b.p. 40–60 °C), and then crystalized from benzene.

*N-(4-(2-(5-Oxo-6-(3-oxocyclopentyl)-2-phenyl-5,6-dihydroimidazo[2,1-b][1,3,4]thiadiazol-6-yl)acetyl)phenyl)acetamide* (**7a**). Yield 1.57 g (65%), white finely crystalline powder, m.p. 176–178 °C. IR (*KBr*) υ, cm^−1^: 3245 (NH); 1685, 1670, 1650 (CO), 1613 (C=N); ^1^H-NMR (DMSO-*d*_6_), δ, ppm, (*J*, Hz): 1.12 (6H, m, 3CH_2_), 2.02 (1H, dd, *H*-cyclopent.); 2.36 (3H, s, CH_3_); 2.50 (2H, s, CH_2_CO); 7.44–7.73 (9ArH, m, aromatic protons); 13.2 (1H, s, acidic NH proton which exchanged in D_2_O) and found, %: C 63.25; H 4.65, N 11.79, S 6.77 for C_25_H_22_N_4_SO_4_. Calculated, %: C 63.28, H 4.67, N 11.81, S 6.76. MS: *m*/*z* 474; 432 [M^+^ − CH2=C=O]; 141 [imidazolothiadiazole moiety].

*N-(4-(2-(2-(4-Chlorophenyl)-5-oxo-6-(3-oxocyclopentyl)-5,6-dihydroimidazo[2,1-b][1,3,4]thiadiazol-6-yl)acetyl)phenyl)acetamide* (**7b**). Yield 1.88 g (74%), white finely crystalline. m.p. 210–212 °C. IR (KBr), υ, cm^−1^: 3245 (NH); 1710, 1691, 1655 (CO); 1630 (C=N); ^1^H-NMR (DMSO-*d*_6_), δ, ppm (*J*, Hz): 1.2 (6H, m, 3CH_2_), 2.02 (1H, dd, *H*-cyclopent.); 2.47 (3H, s, CH_3_); 2.51(2H, s, CH_2_CO); 7.44–7.83 (8ArH, m, aromatic protons; 8.2 (1H, s, acidic NH proton which exchanged in D_2_O); ^13^C-NMR (DMSO), δ, ppm, 22.4 (CH_3_CO); 31.8 (C_3,4_ cyclopent); 57.2 (C_5_ cyclopent); 67.8 (CH_2_CO spiro); 109.3 (C_2_, CH, cyclopent); 119.6 (C_3,5_ Ar^−^); 122.2 (C spiro); 124.3 (C_3,5_ Ar); 131.7 (C_2,6_ Ar^−^); 135.5 (C_2,6_ Ar); 141.5 (C_4_ Ar^−^), 143.4 (C_4_ Ar), 144.1 (C_1_ Ar^−^), 152.6 (C_1_ Ar), 161.0 (CNS), 164.0 (CN_2_S), 167.0 (CO imidaz), 168.0 (CO amide), 190.2 (CO ketone) 192.0 (CO cyclopent) and found, %: C 59.05, H 4.15, N 11.03, Cl 6.94, S 6.28 for C_25_H_21_N_4_O_4_ClS. Calculated, %: C 59.00, H 4.16, N 11.01, Cl 6.96, S 6.30.

*6-(2-(4-Bromophenyl)-2-oxoethyl)-2-(4-chlorophenyl)-6-(3-oxocyclopentyl)imidazo[2,1-b][1,3,4]thiadiazol-5(6H)-one* (**7c**). Yield 1.83 g (70%), white finely crystalline. m.p. 196–198 °C. IR (KBr), υ, cm^−1^: 3245 (NH); 1710, 1691, 1655 (CO); 1630 (C=N). ^1^H-NMR (DMSO-*d*_6_), δ, ppm, (*J*, Hz): 1.2 (6H, m, 3CH_2_), 2.08 (1H, dd, *H*-cyclohex.), 2.32 (3H, s, CH_3_), 2.53 (2H, s, CH_2_CO), 7.62–7.83 (8ArH, m, aromatic protons), 8.2 (1H, s, acidic NH proton which exchanged in D_2_O); And found, %: C 52.05; H 3.25; N 7.93, Br 15.03, Cl 6.66, S 6.00 for C_23_H_17_N_3_O_3_BrClS. Calculated, %: C 52.04, H 3.23; N 7.92, Br 15.05, Cl 6.68, S 6.03; MS: *m*/*z* 531 [M^+^ + 2], 529 [M^+^] 170; 139.

### 3.7. General Procedure for Synthesis of the Compounds ***8a***–***c***

A mixture of **7a** (1.2 g, 2.5 mmol) and acetic anhydride (5 mL, 50 mmol) was refluxed in water bath for 2 h. The excess acetic anhydride was removed by fraction distillation and the separated product was filtered, dried and recrystallized from a mixture of toluene–ethanol.

*N-(4-(5′-Oxo-2′-phenyl-6,7-dihydro-5H,5′H-spiro[cyclopenta[b]pyran-4,6′-imidazo[2,1-b][1,3,4]thiadiazol]-2-yl)phenyl)acetamide* (**8a**). Yield 502 mg (45%), white powder. m.p. 140–142 °C. IR (KBr), υ, cm^−1^: 3245 (NH); 1646, 1668 (CO); 1613 (C=N). ^1^H-NMR (DMSO-*d*_6_), δ, ppm (*J*, Hz): 1.43 (6H, m, 3CH_2_), 2.5 (3H, s, CH_3_), 6.7 (1H, s, pyrane-H), 7.44–7.73 (9ArH, m, aromatic protons); 13.2 (1H, s, acidic NH proton which exchanged in D_2_O), and found, %: C 65.75, H 4.40, N 12.25, S 7.00 for C_25_H_20_N_4_O_3_S. Calculated, %: C 65.77, H 4.42, N 12.27, S 7.02; MS: *m*/*z* 456 [M^+^], 337 [M^+^ − PhNCO], 141 [imidazolothiadiazole moiety].

*N-(4-(2′-(4-Chlorophenyl)-5′-oxo-6,7-dihydro-5H,5′H-spiro[cyclopenta[b]pyran-4,6′-imidazo[2,1-b][1,3,4]thiadiazol]-2-yl)phenyl)acetamide* (**8b**). Yield 516 mg (39%), white powder. m.p. 172–174 °C. IR (KBr) υ, cm^−1^: 1630 (C=N), 1645, 1670 (CO), 3245 (NH). ^1^H-NMR (DMSO-*d*_6_), δ, ppm, (*J*, Hz): 1.37 (6H, m, 3CH_2_); 2.06 (3H, s, CH_3_), 6.6–6.7 (s,1H, pyrane), 7.44–7.83 (8ArH, m, aromatic protons), 8.2 (1H, s, acidic NH proton which exchanged in D_2_O), found, %: C 61.13, H 3.88, N 11.43, Cl 7.20, S 6.50 for C_25_H_19_N_4_O_3_ClS. Calculated, %: C 61.16, H 3.90, N 11.41, Cl 7.22, S 6.53; MS: *m*/*z* 490 [M^+^], 377 [M^+^ − PhCl], 170, 140.

*2-(4-Bromophenyl)-2′-(4-chlorophenyl)-5′-oxo-6,7-dihydro-5H,5′H-spiro[cyclopenta[b]pyran-4,6′-imidazo[2,1-b][1,3,4]thiadiazol]-5′-one* (**8c**). Yield 420 mg (35%), white, finely crystalline powder; m.p. 236–238 °C. IR (*KBr*), υ, cm^−1^: 3245 (NH), 1672 (CO), 1630 (C=N); ^1^H-NMR (DMSO-*d*_6_), δ, ppm (*J*, Hz): 1.28 (8H, m, 4CH_2_), 6.6–6.7 (1H, s, pyrane), 7.44–7.83 (8ArH , m, aromatic protons), and found, %: C 53.85, H 2.95; N 8.19, Br 15.56, Cl 6.90, S 6.23 for C_23_H_15_N_3_O_2_BrClS. Calculated, %: C 53.87, H 2.95, N 8.19, Br 15.58, Cl 6.91, S 6.26; MS: *m*/*z* 511 [M^+^].

### 3.8. General Procedure for Synthesis of the Compounds ***9a***–***c***

A mixture of **4a** (3.91 g, 0.01mol), and carbon acids e.g. ethylacetoacetate, ethylcyanoacetate, diethylmalonate, acetylacetone, malononitrile (0.01 mol), (50%) NaOH (8 mL) and ethanol (50 mL), was made and left overnight for 3 days. The reaction mixture was poured into ice/HCl, the crude product was filtered and washed by petroleum ether (b.p. 40–60 °C), and then crystallized from ethanol.

*Ethyl 2-(6-(2-(4-acetamidophenyl)-2-oxoethyl)-5-oxo-2-phenyl-5,6-dihydroimidazo[2,1-b][1,3,4]thiadiazol-6-yl)-3-oxobutanoate* (**9a**). Yield 2.08 g (40%), white finely crystalline, m.p. 180–182 °C. IR (KBr), υ, cm^−1^: 3245 (NH), 1742, 1671, 1655 (CO), 1630 (C=N). ^1^HNMR (DMSO-*d*_6_), δ, ppm, (*J*, Hz): 1.21 (3H, t, CH_3_), 2.32–2.34 (6H, s, 2CH_3_), 2.47 (2H, s, CH_2_CO), 4.23 (2H, q, CH_2_CO), 5.3 (1H, s, methine), 7.62–7.83 (9ArH, m, aromatic protons), 11.2 (1H, s, acidic NH proton which exchanged in D_2_O); and found, %: C 59.93, H 4.62, N 10.72, S 6.14 for C_26_H_24_N_4_O_6_S. Calculated, %: C 59.99, H 4.65, N 10.76, S 6.16; MS: *m*/*z* 520 [M^+^], 170, 139.

*Ethyl 2-(6-(2-(4-acetamidophenyl)-2-oxoethyl)-5-oxo-2-phenyl-5,6-dihydroimidazo[2,1-b][1,3,4]thiadiazol-6-yl)-2-cyanoacetate* (**9b**). Yield 1.87 g (37%), white finely crystalline, m.p. 164–166 °C. IR (KBr), υ, cm^−1^: 3331, 3245 (NH), 1734, 1670, 1645 (CO), 1630 (C=N). ^1^H-NMR (DMSO-*d*_6_), δ, ppm, (*J*, Hz): 1.23 (3H, t, CH_3_), 2.32 (3H, s, CH_3_), 2.47 (2H, s, CH_2_CO), 4.09 (2H, q, CH_2_CO), 5.1 (1H, s, methine), 7.35–7.72 (9ArH, m, aromatic protons), 12.1 (1H, s, acidic NH proton which exchanged in D_2_O), found, %: C 59.60, H 4.17, N 13.88, S 6.35 for C_25_H_21_N_5_O_5_S. Calculated, %: C 59.63, H 4.20, N 13.91, S 6.37; MS: *m*/*z* 503 [M^+^], 430 [M^+^ − COOEt], 170, 140.

*Diethyl 2-(6-(2-(4-acetamidophenyl)-2-oxoethyl)-5-oxo-2-phenyl-5,6-dihydroimidazo[2,1-b][1,3,4]thiadiazol-6-yl)malonate* (**9c**). Yield 3.86 g (70%), white finely crystalline. m.p. 144–146 °C. IR (KBr), υ, cm^−1^: 3245 (NH), 1671, 1742 (CO), 1630 (C=N). ^1^H-NMR (DMSO-*d*_6_), δ, ppm, (*J*, Hz): 1.22 (6H, t, 2CH_3_), 2.32–2.35 (6H, s, 2CH_3_), 2.47 (2H, s, CH_2_CO), 4.45 (4H, q, CH_2_CO), 5.6 (1H, s, methine), 7.62–7.83 (9ArH, m, aromatic protons), 11.2 (1H, s, acidic NH proton which exchanged in D_2_O). ^13^C-NMR (DMSO), δ, 14.5 (2CH_3_CH_2_), 22.4 (CH_3_CO Ar), 60.5 (2CH_3_CH_2_CO), 64.7 (CH_2_CO spiro), 92.6 (CH(CO)_2_), 114.6 (C_4_ Ph), 122.1 (C_3,5_ Ph), 127.5 (C spiro), 128.2 (C_3,5_ Ar), 129.6 (C_2,6_ Ph), 131.6 (C_2,6_ Ar), 134.2 (C_4_ Ar), 137.9 (C_1_ Ph), 140.2 (C_1_ Ar), 149.5 (CNS), 154.1 (CN_2_S), 163.4 (CO imidaz), 168.3 (CO amide), 176.6 (2CO ester), 193.0 (CO ketone), and found, %: C 58.86; H 4.72; N 10.15, S 5.79 for C_27_H_26_N_4_O_7_S. Calculated, %: C 58.90, H 4.76, N 10.18, S 5.82; MS: *m*/*z* 550 [M^+^], 170, 139.

*N-(4-(2-(6-(2,4-Dioxopentan-3-yl)-5-oxo-2-phenyl-5,6-dihydroimidazo[2,1-b][1,3,4]thiadiazol-6-yl)acetyl)phenyl)acetamide* (**9d**). Obtained similarly to compound **9a**, from compound **4a** (3.91 g, 0.01 mol) and acetylacetone (1.05 mL, 0.01 mol). Crystalised from benz-ethanol. Yield 2.94 g (60%), white powder. m.p. 158–160 °C. IR (KBr) υ, cm^−1^: 3245 (NH); 1670, 1645 (CO), 1630 (C=N). ^1^H-NMR (DMSO-*d*_6_), δ, ppm (*J*, Hz): 2.32–2.46 (9H, br. s, 3CH_3_), 2.47 (2H, s, CH_2_CO), 5.2 (1H, s, methine), 7.35–7.72 (9ArH, m, aromatic protons), 12.1 (1H, s, acidic NH proton which exchanged in D_2_O), and found, %: C 61.22, H 4.49, N 11.40, S 6.52 for C_25_H_22_N_4_O_5_S. Calculated, %: C 61.21; H 4.52; N 11.42, S 6.54; MS: *m*/*z* 490 [M^+^]; 447 [M^+^ − COCH_3_], 170, 140.

*N-(4-(2-(6-(Dicyanomethyl)-5-oxo-2-phenyl-5,6-dihydroimidazo[2,1-b][1,3,4]thiadiazol-6-yl)acetyl)phenyl)acetamide* (**9e**). Crystalized from ethanol. Yield 3.29 g (72%), white powder, m.p. 122–124 °C. IR (KBr), υ, cm^−1^: 3320 (NH); 1655 (CO); 1630 (C=N). ^1^H-NMR (DMSO-*d*_6_), δ, ppm, (*J*, Hz): 2.32 (3H, s, CH_3_), 2.47 (2H, s, CH_2_CO), 5.0(1H, s, methine), 7.35–7.72 (9ArH, m, aromatic protons), 12.1 (1H, s, acidic NH proton which exchanged in D_2_O). ^13^C-NMR, δ, 21.8 (CH_3_CO Ar), 62.5 (CH_2_CO spiro), 95.1 (CH(CN)_2_), 112.6 (C_4_ Ph), 119.3 (C_3,5_ Ph), 124.5 (C spiro), 125.4 (C_3,5_ Ar), 128.6 (C_2,6_ Ph), 131.2 (C_2,6_ Ar), 133.4 (C_4_ Ar), 135.6 (C_1_ Ph), 139.2 (C_1_ Ar), 147.7 (CNS), 152.1 (CN_2_S), 163.2 (CO imidaz), 167.3 (CO amide), 170.6 (2CN), 189.4 (CO ketone); found, %: C 60.49, H 3.50, N 18.38, S 7.00 for C_23_H_16_N_6_O_3_S: C 60.52, H 3.53, N 18.41, S 7.02; and MS: *m*/*z* 456 [M^+^], 380 [M^+^ − Ph], 170, 140.

### 3.9. General Procedure for Synthesis of the Compounds ***10a***–***c*** and ***11a***–***d***

The adduct **9** (1.9 mmol) was fused in oil bath for 1 h. The reaction mixture was poured into ice, the crude product filtered and washed by petroleum ether (b.p. 40–60 °C), and then crystalized.

*N-(4-(*3′*-Acetyl-2′,5-dioxo-2-phenyl-2′,3′-dihydro-5H-spiro[imidazo[2,1-b][1,3,4]thiadiazole-6,4′-pyran]-6′-yl)phenyl)acetamide* (**10a**). Crystalized from dioxane. Yield 495 mg (55%), white finely crystalline, m.p. 210–212 °C. IR (KBr), υ, cm^−1^: 3245 (NH), 1743, 1685, 1670, 1650 (CO), 1613 (C=N); ^1^H-NMR (DMSO-*d*_6_), δ, ppm, (*J*, Hz): 2.36 (6H, s, 2CH_3_), 4.50 (1H, s, CH(CO)_2_), 6.2 (1H, s, PyH), 7.44–7.73 (9ArH, m, aromatic protons), 13.2 (1H, s, acidic NH proton which exchanged in D_2_O); found: %: C 60.70, H 3.79, N 11.75, S 6.76 for C_24_H_18_N_4_O_5_S. Calculated, %: C 60.75, H 3.82, N 11.81, S 6.76; MS: *m*/*z* 474 [M^+^], 141 [imidazolothiadiazole moiety].

*N-(4-(3′-Cyano-2′,5-dioxo-2-phenyl-2′,3′-dihydro-5H-spiro[imidazo[2,1-b][1,3,4]thiadiazole-6,4′-pyran]-6′-yl)phenyl)acetamide* (**10b**). Crystalized from ethanol. Yield 409 mg (45%), white finely crystalline, m.p. 198–200 °C. IR (KBr), υ, cm^−1^: 3245 (NH), 2220 (CN), 1740, 1691, 1672, 1655, (CO), 1630 (C=N); ^1^H-NMR (DMSO-*d*_6_), δ, ppm, (*J*, Hz): 2.47 (3H, s, CH_3_), 4.51 (1H, s, CH(CO)(CN)), 6.1 (1H, s, PyH), 7.44–7.83 (9ArH, m, aromatic protons), 8.2 (1H, s, acidic NH); found, %: C 60.35, H 3.28, N 15.31, S 7.00 for C_23_H_15_N_5_O_4_S. Calculated, %: C 60.39; H 3.31; N 15.31, S 7.01.

*Ethyl 6′-(4-acetamidophenyl)-2′,5-dioxo-2-phenyl-2′,3′-dihydro-5H-spiro[imidazo[2,1-b][1,3,4] thiadiazole-6,4′-pyran]-3′-carboxylate* (**10c**). Yield 1.17 g (85%), white finely crystalline, m.p. 162–164 °C. IR (KBr), υ, cm^−1^: 3245 (NH), 1752, 1738, 1670, 1650 (CO), 1613 (C=N). ^1^H-NMR (DMSO-*d*_6_), δ, ppm (*J*, Hz): 1.2 (2H, t, CH_2_), 2.5 (3H, s, CH_3_), 4.11 (2H, q, CH_2_O), 4.4 (1H, s, CH(CO)_2_), 6.2(1H, s, PyH), 7.44–7.73 (9ArH, m, aromatic protons); 13.2 (1H, s, acidic NH proton which exchanged in D_2_O); found, %: C 59.50, H 3.95, N 11.08, S 6.32 for C_25_H_20_N_4_O_6_S. Calculated, %: C 59.52, H 4.00, N 11.11, S 6.35; MS: *m*/*z* 504 [M^+^], 462 [M^+^ − CH2=C=O], 141.

*Ethyl 6′-(4-acetamidophenyl)-2′-methyl-5-oxo-2-phenyl-5H-spiro[imidazo[2,1-b][1,3,4]thiadiazole-6,4′-pyran]-3′-carboxylate* (**11a**). Crystalized from benzene. Yield 219 mg (23%), white powder, m.p. 180–182 °C. IR (KBr), υ, cm^−1^: 3245 (NH), 1742, 1671, 1655, (CO), 1630 (C=N); ^1^H-NMR (DMSO-*d*_6_), δ, ppm, (*J*, Hz): 1.21 (3H, t, CH_3_), 2.32 (6H, s, 2CH_3_), 4.23 (2H, q, CH_2_CO), 6.3 (s, 1H, PyH), 7.62–7.83 (9ArH, m, aromatic protons), 11.2 (1H, s, acidic NH proton which exchanged in D_2_O); found, %: C 62.15, H 4.38, N 11.13, S 6.35 for C_26_H_22_N_4_O_5_S. Calculated, %: C 62.14, H 4.41, N 11.15, S 6.38; MS: *m*/*z* 502 [M^+^], 170, 139.

*Ethyl 6′-(4-acetamidophenyl)-2′-amino-5-oxo-2-phenyl-5H-spiro[imidazo[2,1-b][1,3,4]*thiadiazole*-6,4′-pyran]-3′-carboxylate* (**11b**). Crystalized from benzene. Yield 249 mg (25%), white powder, m.p. 164–166 °C. IR (KBr), υ, cm^−1^: 3331, 3245 (NH), 1734, 1670, 1645 (CO), 1630 (C=N); ^1^H-NMR (DMSO-*d*_6_), δ, ppm, (*J*, Hz): 1.23 (3H, t, CH_3_), 2.32 (6H, s, 2CH_3_), 4.09 (2H, q, CH_2_CO), 5.2(2H, br. s, NH_2_), 6.1 (1H, s, PyH), 7.35–7.72 (9ArH, m, aromatic protons), 12.1 (1H, s, acidic NH proton which exchanged in D_2_O), and found, %: C 59.60, H 4.18, N 13.90, S 6.35 for C_25_H_21_N_5_O_5_S. Calculated, %: C 59.63, H 4.20, N 13.91, S 6.37; MS: *m*/*z* 503 [M^+^], 430 [M^+^ − COOEt]; 170. 

*N-(4-(3′-Acetyl-2′-methyl-5-oxo-2-phenyl-5H-spiro[imidazo[2,1-b][1,3,4]thiadiazole-6,4′-pyran]-6′-yl)phenyl)acetamide* (**11c**). Yield 810 mg (64%), white powder; m.p. 194–196 °C. IR (KBr), υ, cm^−1^: 3245(NH), 1687, 1670, 1650, (CO), 1613 (C=N). ^1^H-NMR (DMSO-*d*_6_), δ, ppm, (*J*, Hz): 2.52 (9H, br. s, 3CH_3_), 6.3 (1H, s, PyH), 7.35–7.80 (9ArH, m, aromatic protons), 11.6 (1H, s, acidic NH proton which exchanged in D_2_O); found, %: C 63.52, H 4.25, N 11.85, S 6.75 for C_25_H_20_N_4_O_4_S. Calculated, %: C 63.55, H 4.27, N 11.86, S 6.78; MS: *m*/*z* 472 [M^+^], 353 [M^+^ − PhN=C=O], 141 [imidazolothiadiazole moiety].

*N-(4-(2′-Amino-3′-cyano-5-oxo-2-phenyl-5H-spiro[imidazo[2,1-b][1,3,4]thiadiazole-6,4′-pyran]-6′-yl)phenyl)acetamide* (**11d**). Yield 600 mg (60%), white finely crystalline, m.p. 222–224 °C. IR (KBr), υ, cm^−1^: 3285, 3245 (NH), 2220 (CN), 1672, 1655 (CO), 1630 (C=N); ^1^H-NMR (DMSO-*d*_6_), δ, ppm, (*J*, Hz): 2.46 (3H, s, CH_3_), 5.2 (2H, br. s, NH_2_), 6.3 (1H, s, PyH), 7.25–7.66 (9ArH, m, aromatic protons), 11.3 (1H, s, acidic NH proton which exchanged in D_2_O); found, %: C 60.50; H 3.51; N 18.40, S 7.00 for C_23_H_16_N_6_O_3_S. Calculated, %: C 60.52, H 3.53, N 18.41, S 7.02. MS: *m*/*z* 456 [M^+^]. 

## 4. Conclusions

In the present work, a series of novel chalcone and the spiro heterocyclic derivatives **4**–**11** were synthesized using 4-Aryl-4-oxo-2-butenoic acids **1a**–**b** as starting materials. The structures of the new compounds were elucidated using IR, ^1^H-NMR, ^13^C-NMR and mass spectroscopy. Some of the newly synthesized compounds were screened against bacterial strains and most of them showed high antibacterial activities that were confirmed by QSAR study. Electron-withdrawing substituents are lower the HOMO energy, and increase (κ2 index) represents a positive contribution to the antibacterial activity.

## References

[1] Vitorovic-Todorovi M.D., Eric-Nikoli A., Kolundzija B., Hamel E., Risti S., Jurani I.O., Drakuli B.J. (2013). (*E*)-4-Aryl-4-oxo-2-butenoic acid amides, chalconeearoylacrylic acid chimeras: Design, antiproliferative activity and inhibition of tubulin polymerization. Eur. J. Med. Chem..

[2] Dimmock J.R., Elias D.W., Beazely M.A., Kandepu N.M. (1999). Bioactivites of Chalcones. Curr. Med. Chem..

[3] Carlo G.D., Mascolo N., Izzo A.A., Capasso F. (1999). Flavonoids: Old and new aspects of a class of natural therapeutic drugs. Life Sci..

[4] Hwang K., Kim H., Han I., Kim B. (2012). Synthesis of heterocyclic chalcone derivatives and their radical scavenging ability toward 2,2-diphenyl-1-picrylhydrazyl (DPPH) free radicals. Bull. Korean Chem. Soc..

[5] Tran T., Nguyen T., Do T., Huynh T., Tran C., Thai K. (2012). Synthesis and antibacterial activity of some heterocyclic chalcone analogues alone and in combination with antibiotics. Molecules.

[6] Saini R.K., Kumari N., Joshi Y.C., Joshi P., Shekhawat S.S. (2007). Solvent free microwave assisted synthesis of chalcones and their antifungal activities. Asian J. Chem..

[7] Toshihiro S.M., Minoru H., Yuji T., Toshiyuki S., Takunobu O., Osamu N., Katsumasa K., Hidefumi H., Masayasu G., Akira I. (2009). Identification of novel and orally active spiroindoline NPY Y5 receptor antagonists. Bioorg. Med. Chem. Lett..

[8] Nagaaki S., Makoto J., Shiho I., Keita N., Hiroyasu T., Makoto A., Osamu O., Hisashi I., Akira G., Akane I. (2009). Discovery of substituted 2,4,4-triarylimidazoline derivatives as potent and selective neuropeptide YY5 receptor antagonists. Bioorg. Med. Chem. Lett..

[9] Laragh J.H., Sealey J.E. (2001). K^+^ depletion and the progression of hypertensive disease or heart failure the pathogenic role of diuretic-induced aldosterone secretion. Hypertension.

[10] Laragh J.H. (2001). Laragh’s lessons in pathophysiology and clinical pearls for treating hypertension. Am. J. Hypertens..

[11] Epstein M. (2001). Aldosterone as a mediator of progressive renal disease: Pathogenetic and clinical implications. Am. J. Kidney Dis..

[12] Hurwitz A., Ruhl C.E., Kimler B.F., Topp E.M., Mayo M.S. (2003). Gastric function in the elderly: Effects on absorption of ketoconazole. J. Clin. Pharmacol..

[13] Dariusz A.P., Andrzej M.B., Agnieszk E.L., Bohdan J.S., Jerzy K. (2007). Synthesis of 1-[4-[4-(adamant-1-yl)phenoxymethyl]-2-(4-bromophenyl)-1,3-dioxolan-2-yl-methyl]imidazole with expected antifungal and antibacterial activity. Acta Pol. Pharm. Drug Res..

[14] Prabodh C.S., Sunil V.S., Sandeep J., Dalbir S., Bhojraj S. (2009). Synthesis of some new isoxazoline derivatives as possible anti-candida agents. Acta Pol. Pharm. Drug Res..

[15] Marcin M., Michal Z., Magdalena T., Stanislaw R. (2008). Synthesis, immunological activity and computational study of 5-amino-3-methyl-4-isoxazole-carboxylic acid semicarbazides and thiosemicarbazides. Acta Pol. Pharm. Drug Res..

[16] Justyna Q., Dorota O., Zofia Z., Ewa A., Lucjusz Z. (2008). Synthesis of 2,3-dihydro-7-nitro-imidazo[5,1-*b*]oxazole as potential tuberculostatic agents. Acta Pol. Pharm. Drug Res..

[17] Rizk S., EL-Hashash M., Aburzeza M. (2011). 1.4-Arylation of β-(4-acetylaminobenzoyl)acrylic acid with activated aromatic hydrocarbons under fridel-crafts conditions and some studies with the products. Egypt J. Chem..

[18] El-Hashash M., Rizk S., Aburzeza M. (2011). Utility of p-acetamidobenzoyl prop-2enoic acid in the synthesis of new α-amino acids and using them as building blocks in heterocyclic synthesis. Egypt J. Chem..

[19] Rizk S.A. (2011). Utility of *E*-1-(4-acetamidobenzoyl)-2-oxirane carboxylic acid in synthesis some fused heterocycles and spiro compounds. Amm. J. Chem..

[20] Rizk S., El-Hashash M. (2011). 2-(3,4-Dimethylphenyl-3-(3,4-dichloro(3,4-dimethyl)) benzoyl ) propanoic acids as precursors in the synthesis of some heterocyclic compounds. Egypt J. Chem..

[21] Rizk S., El-Hashash M., Mostafa K. (2008). Utility of β-aroyl acrylic acids in heterocyclic synthesis. Egypt J. Chem..

[22] El-Kadi M., El-Hashash M., Sayed M. (1981). Action of Hydrazine, amine and thiourea upon 3-(4-chloro-3-*methyl*)benzoyl acrylic acid. Rev. Roum. Chim..

[23] El-Hashash M., El-Kady M. (1978). Some reactions of 3-aroyl acrylic acid epoxides. Rev. Roum. Chim..

[24] Prasad Y.R., Kumar P.R., Smiles D.J., Babub P.A. (2008). QSAR studies on chalcone derivatives as antibacterial agents. Arkivoc.

[25] Azab M.E., Rizk S.A., Amr A.E. (2015). Synthesis of some novel heterocyclic and schiff base derivatives as antimicrobial agents. Molecules.

[26] Tatyan L.P., Fedor G.Y., Victoria V.L., Svetlana V.S., Oleg V.S., Vladimir I.M., Alexander S.K. (2014). The regioselective synthesis of spirooxindolopyrrolidines and pyrrolizidines via three-component reactions of acrylamides and aroylacrylic acids with isatins and α-amino acids. Beilstein J. Org. Chem..

[27] El-Hashash M.A., Rizk S.A. (2015). Regioselective Diastereomeric michael adducts as building blocks in heterocyclic synthesis. J. Heterocycl. Chem..

[28] Venkataraman L., Park Y.S., Whalley A.C., Nuckolls C., Hybertsen M.S., Steigerwald M.L. (2007). Electronics and chemistry: Varying single-molecule junction conductance using chemical substituents. Nano Lett..

[29] Parveen Z., Brunhofer G., Jabeen I., Erker T., Chiba P., Ecker G.F. (2014). Synthesis, biological evaluation and 3D-QSAR studies of new chalcone derivatives as inhibitors of human P-glycoprotein. Bioorg. Med. Chem..

[30] Takayanagri H., Kitano Y., Yano T., Umeki H., Hara H. (1995). Bioisoster of aroyl vinylamide antitumor, and tyrosine kinase inhibition. J. Can. Pat. Appl..

[31] Jaramillo M.C., Mora C., Vélez L.E., Quijano J. (2009). Kinetic and theoretical study of the chalcones as inhibitors of beta-lactamase enzyme. Med. Chem..

[32] Kalagutkar A.S., Naguyen H.T., Vaz Alfin D.N., Dalvu D.K., Mcleod D.G., Murray J.C. (2003). *In vitro* metabolism studies on the isoxazole ring scission in the anti-inflammatory agent leflunomide to its active α-cyanoenol metabolite a771726: Mechanistic similarities with the cytochrome p450-catalyzed dehydration of aldoximes. Drug. Metab. Dispos..

[33] Mitwatashi S., Arikawa Y., Kotani E., Miyamoto M., Naruo K.I., Kimura H., Tanaka T., Asahi S., Ohkawa S. (2005). Novel inhibitor of p38 MAP kinase as an anti-TNF-α drug:  Discovery of *N*-[4-[2-ethyl-4-(3-methylphenyl)-1,3-thiazol-5-yl]-2-pyridyl]benzamide (TAK-715) as a potent and orally active anti-rheumatoid arthritis agent. J. Med. Chem..

[34] Junji I., Yuichiro N., Zhang H., Ryuji U., Kenichi N., Rokuro M., Hiroshi T. (2013). Spirohexalines, new inhibitors of bacterial undecaprenyl pyrophosphate synthase. J. Antibiot..

